# The Long and Short of Next Generation Sequencing for *Cryptosporidium* Research

**DOI:** 10.3389/fcimb.2022.871860

**Published:** 2022-03-28

**Authors:** Tapoka T. Mkandawire, Adam Sateriale

**Affiliations:** Laboratory of Host-Pathogen Interactions in Cryptosporidiosis, Francis Crick Institute, London, United Kingdom

**Keywords:** apicomplexa, long read sequencing, population genomics, gene expression, parasite control

## Abstract

The intestinal parasite *Cryptosporidium* is a significant cause of severe diarrhoeal disease that can have long term effects. Therapeutic options remain limited despite a significant impact on public health, partly due to various challenges in the field of *Cryptosporidium* research, including the availability of genomic and transcriptomic data from environmental and clinical isolates. In this review we explore how long read DNA and RNA sequencing technologies have begun to provide novel insights into the biology of the parasite. The increased deployment of these technologies will help researchers address key gaps in the understanding of *Cryptosporidium* biology, and ultimately drive translational research and better parasite control.

## Introduction

*Cryptosporidium* is an intracellular parasite that is an important cause of global diarrhoeal disease in animals and humans. Infection in animals, particularly livestock, results in increased agricultural cost and decreased production (Shaw et al., 2020). In humans, children and immunocompromised patients carry a disproportionate burden of disease and early life infections have been shown to be associated with stunted growth (Khalil et al., 2018). Acute disease accounts for 4.22 million disability adjusted life years (DALYs) annually, yet the chronic effects of disease, such as growth stunting, are estimated to account for a further 7.85 million DALYs (Khalil et al., 2018). Despite this substantial impact on public health, there are no fully effective treatments and no available vaccinations against disease (Schneider et al., 2021).

The life cycle of the *Cryptosporidium* parasite is complex and progresses through several morphologies – the infective form of the parasite is the oocyst, which is an environmentally hardy form that transmits *via* a faecal-oral route. Oocysts that are ingested by the host release four motile forms of the parasite, sporozoites, that invade epithelial cells that line the host intestinal tract. Once inside the epithelial cell, the parasite undergoes asexual replication, known as schizogony or merogony, before making a sexual commitment and undergoing sexual replication, known as gametogony. These sexual forms then come together to create new infective oocysts that are released into the environment in the host faeces (Current and Reese, 1986).

*Cryptosporidium* is a prominent infection in vertebrates and there are over 35 recognised species with varying host specificity (Feng et al., 2018). Infection in humans is primarily driven by two species: *Cryptosporidium hominis* and *Cryptosporidium parvum.* To date, fifteen unique genome assemblies of *Cryptosporidium* have been generated and eight genomes annotated (Warrenfeltz et al., 2020). The inconsistent availability and quality of *Cryptosporidium* reference genomes has not only impacted our understanding of the basic biology of the parasite, but translational advances in surveillance, diagnostics, and therapeutics have also been lagging. For many *Cryptosporidium* genomes significant time has elapsed since they were initially sequenced and assembled. These references may require updated sequencing with next generation technologies, or genome re–annotation and polishing to ensure that as reference genomes they do not mislead interpretations during genomic or molecular studies (Baptista et al., 2022). A greater understanding of the parasite genome *in silico* will complement recent *in vivo* and *in vitro* advances that have increased our understanding of parasite and infection biology (Marzook and Sateriale, 2020). In this review we describe some of the many challenges facing the *Cryptosporidium* research community, and we explore the ways recent advances in sequencing, specifically long read technologies, have begun and will continue to address them.

## Challenges in *Cryptosporidium* Research

Historically, *Cryptosporidium* research has suffered from a lack of suitable models to study infection. However, recent developments have started to turn the tides and expand the capabilities of researchers. Stem-cell based models of infection, such as the organoid (Heo et al., 2018) and air-liquid interface models (Wilke et al., 2019), more closely recapitulate the cellular diversity and architecture of the gut, allowing for more translational studies of infection. A recently developed mouse model of cryptosporidiosis also offers a fully genetically tractable system that replicates human pathology *in vivo* (Sateriale et al., 2019). Despite these advances, there is still no reliable method for continuous culture of the *Cryptosporidium* parasite. *In vitro* culturing is still limited to pre-fertilisation stages as parasites are unable to complete sexual reproduction using traditional cell culture methods (Tandel et al., 2019). These culturing constraints mean that clonal populations of parasites cannot be generated, and this is particularly pertinent for genomic studies.

The *Cryptosporidium* genome is very compact at ~9 Mb and the parasite scavenges nutrients from the host, obscuring metabolic pathways. The parasite also uses alternative splice forms, making it a challenge to validate and annotate genes and their functions (Baptista et al., 2022). As a result, many genome assemblies contain large percentages of hypothetical or uncharacterised proteins (**Table 1**). Oocysts are often sampled as the most readily available life cycle stage, however they only display minute morphological differences at the species level. Individuals in endemic regions are often co-infected with multiple strains or species and these differences can only be observed genetically (Ryan et al., 2021). Importantly, as each oocyst contains four sporozoites, an individual oocyst is considered a mixed population. Even single cell sequencing only partially addresses concerns around mixed populations, but does facilitate the detection of low frequency variation (Troell et al., 2016; Baptista et al., 2021). Genetic recombination occurs during sexual replication generating diversity that influences virulence and transmissibility. Establishing clonal cultures of isolates would address some of the challenges in deconvoluting this diversity.

**Table 1:** Genome assembly statistics for *Cryptosporidium* species.

	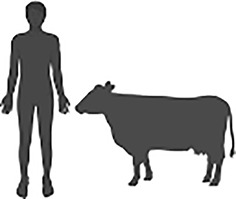	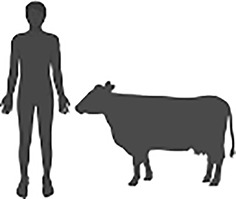	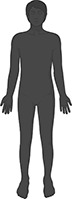	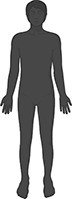	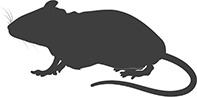	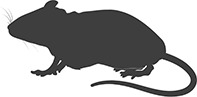	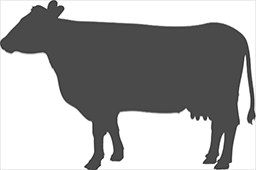	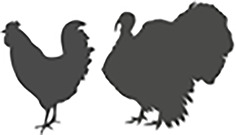	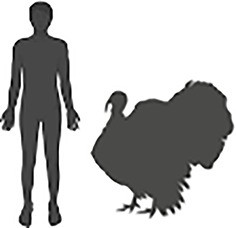	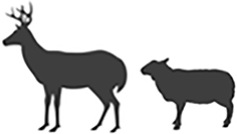
	*Cryptosporidium parvum*	*Cryptosporidium parvum*	*Cryptosporidium hominis*	*Cryptosporidium hominis*	*Cryptosporidium tyzzeri*	*Cryptosporidium muris*	*Cryptosporidium andersoni*	^*^ *Cryptosporidium baileyi*	*Cryptosporidium meleagridis*	*Cryptosporidium ubiquitum*
	IOWA-ATCC	Iowa II	isolate 30976	TU502	isolate UGA55	RN66	isolate 30847	TAMU-09Q1	strain UKMEL1	isolate 39726
Total sequence length (bases)	9,122,263	9,102,324	9,059,225	8,915,516	9,015,711	9,245,251	9,088,557	8,493,640	8,973,200	8,970,213
Number of contigs^1^	1	18	53	365	3	97	205	145	57	63
Contig N50 (bases)^2^	1,108,396	1,014,526	364,413	48,000	1,108,290	520,347	124,036	203,018	322,908	310,873
Total number of chromosomes and plasmids^3^	8	8	0	0	8	0	0	0	0	0
% of hypothetical genes^4^	33	40	34	60	34	54	55	–	38	59
					Above average		Average		Below average	

^*^Cryptosporidium baileyi genome is unannotated.

^1^Contiguous genomes are arranged in fewer contigs. **Above average <10 contigs.**

^2^Contiguous genomes have a contig N50 of at least 1Mb. **Above average >1 Mb**

^3^Chromosome scale assemblies are more polished. **Above average >1 chromosomes.**

^4^Functional annotation quality can be inferred from the percentage of hypothetical proteins. **Above average <20%.**

## Long Read Genomics to Improve Our Understanding of Parasite Virulence and Transmission

**Figure 1:**
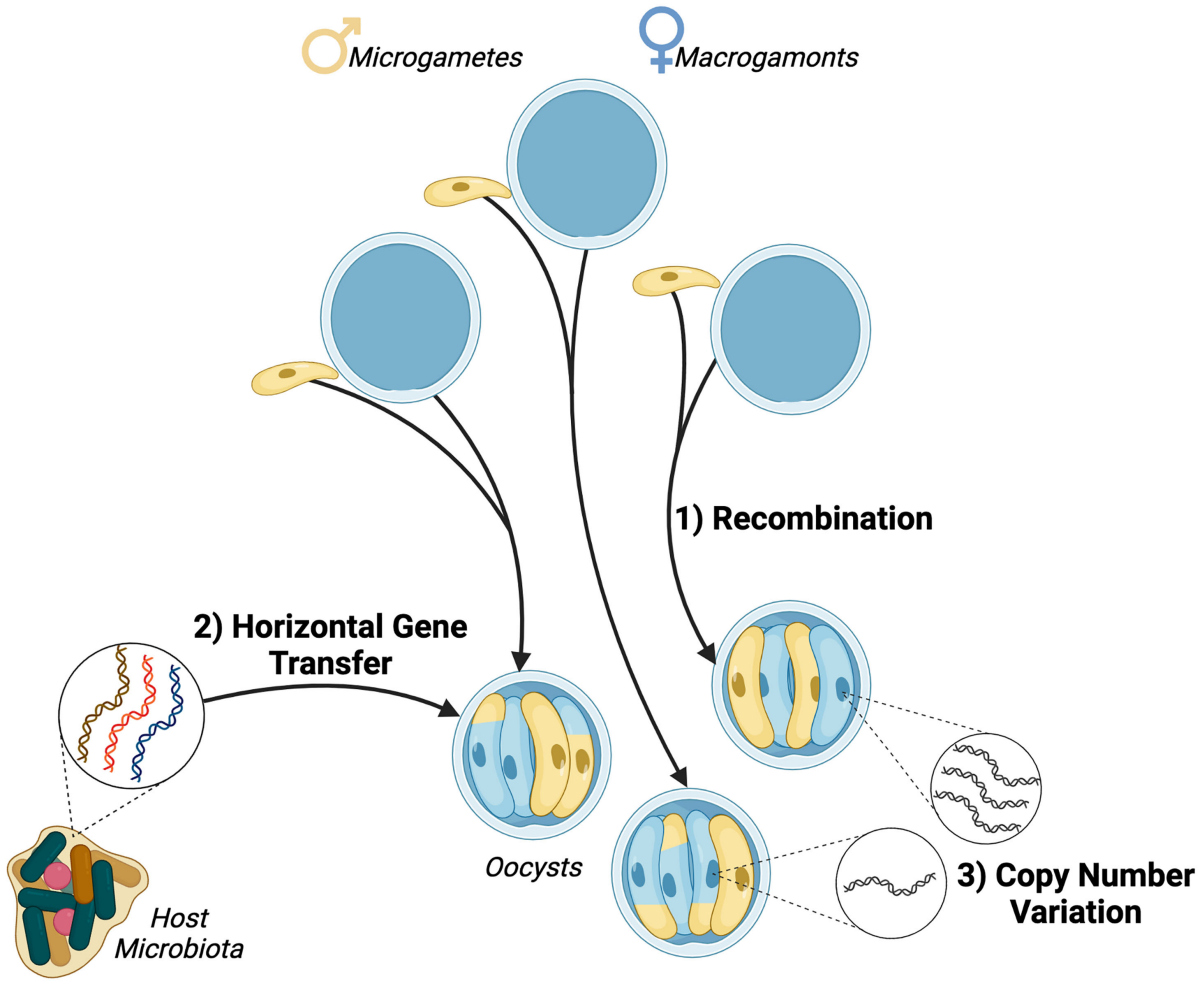
Sources of genetic diversity in *Cryptosporidium* parasites. 1) Recombination occurs during sexual replication and meiotic divisions distribute parental alleles, resulting in recombinant progeny. 2) Horizontal gene transfer of bacterial genes has been observed in *Cryptosporidium* species suggesting a link with the host gastrointestinal microbiota. 3) Copy number variation has been observed in at least 13 *Cryptosporidium parvum* genes with implications for parasite host range and invasion.

Despite the decreasing cost and increasing relative ease of sequencing it is an underutilised tool in *Cryptosporidium* research; and for many years, the field has relied on the *C. parvum* and *C. hominis* genomes generated in 2004 through whole genome shotgun Sanger sequencing and HAPPY mapping (Abrahamsen et al., 2004; Xu et al., 2004). Annotation remains a resource and labour intensive process and while there have been several re-annotations of the *C. parvum* genome, it was only recently updated using a combination of Pacific Biosciences long reads and Illumina DNA and RNA short reads. This combined approach significantly improved the genome assembly quality across key metrics– including contiguity, completeness, and correctness, and generated a high quality *C. parvum* IOWA-ATCC reference genome (Baptista et al., 2022). This revised genome has already revealed several new insights into the parasite’s biology, including the identification of new parasite transporters. One surprising find from this reannotation is the amount of copy number variation in the *Cryptosporidium* genome (**Figure 1**). Variations in copy number have been shown to affect gene expression and increase phenotypic variation (Freeman et al., 2006), and in *C. parvum* it is thought to contribute to phenotypes such as host specificity and sporozoite invasion (Zhang et al., 2019). DNA long reads– especially through intergenic and repeat regions in the genome, can help resolve artificial compression of the genome and reveal variations in copy number (Logsdon et al., 2020). The recent updates to the *C. parvum* genome revealed at least 13 genes with variations in copy number, including the recently identified MEDLE family of host-exported virulence factors (Dumaine et al., 2021). This reannotation of the *C. parvum* genome is an exemplary case and the quality of the *Cryptosporidium* reference genomes varies greatly across species, therefore more concerted efforts are needed to generate high quality references of the other species (**Table 1**). In particular, *C. hominis* as the other prominent causative agent of human disease and *C. tyzzeri* as a murine model of human infection, are both desperately in need of a reannotation. The *C. tyzzeri* genome specifically, illustrates how current strategies using the limited genomic data available can present a false confidence in the quality of a genome assembly. The current *C. tyzzeri* assembly (Sateriale et al., 2019) relies heavily on *C. parvum* data to scaffold the genome and this cross species assembly can lead to a loss of information, especially translocations and inversions. Increased use of long read sequencing across *Cryptosporidium* species will address these inaccuracies and lead to more informative assemblies.

Understanding transmission hinges on the ability to identify and differentiate different genotypes during surveillance. Currently, genes such as *gp60* and 18S rRNA are used for single locus typing (SLT) and a variety of markers are selected on a study by study basis for multilocus sequence typing (MLST) (Robinson and Chalmers, 2012). SLT is not a robust strategy that can account for the effects of recombination during sexual stages. Additionally, the gene currently used for much of single locus typing– *gp60*, is a virulence gene and therefore under selective pressure which may affect its suitability as a marker gene (Morris et al., 2019). Indeed the lack of global geographical sub structuring observed when comparing *gp60* alleles in isolates from different locations demonstrates that the selection pressures driving *gp60* evolution mean it cannot be used for universal single locus typing (Widmer, 2009; Robinson and Chalmers, 2012). In order to perform MLST markers are selected and used in single studies, because when applied to isolates from different geographical regions the performance of these panels declines significantly (Robinson and Chalmers, 2012). Furthermore, isolates identified as the same species through 18S rRNA typing can display differing phenotypes and even different host specificity; together with the knowledge that multiple copies of this gene are present across the genome, this suggests that copy number variation (CNV) or variation at other loci will prove to be more informative (Nader et al., 2019; Baptista et al., 2022). It is important to address these incongruencies moving past our reliance on outdated strategies such as SLT, and developing more broadly applicable MLST panels. Improved genomic sequencing will provide the greater sequence coverage of multiple loci required to fully resolve and understand population.

Another major challenge for the surveillance and population genomics of *Cryptosporidium* is deconvolution. Individuals in endemic regions can present mixed strain and species infections (Ryan et al., 2021) and, without a consensus of marker genes, current strategies struggle to resolve the structure of subpopulations of the same species (Robinson and Chalmers, 2012; Baptista et al., 2021). When investigating subpopulations, the diversity generated by recombination events during sexual replication means that mixed populations of sporozoites can exist in a single oocyst (**Figure 1**) (Dettwiler et al., 2021). Additionally, *Cryptosporidium* spp. have acquired large quantities of genes through horizontal gene transfer (**Figure 1**) (Huang et al., 2004). In order to fully understand the movement of genes into and within parasite populations more high-quality sequences are needed.

## Long Read Transcriptomics to Improve Our Understanding of Parasite Biology and Gene Regulation

Advances in genomics and transcriptomics go hand in hand, and combining long and short read DNA sequencing with short read RNA sequencing (RNAseq) has already improved the annotation of the *C. parvum, C. hominis*, and *C. tyzzeri* genomes (Baptista et al., 2022). Available strand specific RNAseq data has revealed some information regarding splicing mechanisms and isoforms in *Cryptosporidium* (Li et al., 2020). The long non-coding RNAs (lncRNA) identified were predominantly antisense sense transcripts (91.7%) that covered nearly 10% of predicted mRNA transcripts and were primarily located at the 3’ end of the sense mRNA (Li et al., 2020). Additionally, these data provided evidence of splicing in lncRNA and suggested the presence of bidirectional promoters (Li et al., 2020), indicating that the parasite has mechanisms for complex control of gene expression (Gil and Ulitsky, 2018). In organisms with compact genomes, like *Cryptosporidium*, which is only 20% intergenic, utilising RNAseq to annotate and validate genes can be challenging (Baptista et al., 2022). Resolving genes that are in close proximity through transcriptome assembly using short reads can often result in the artificial fusion of exons (Xie et al., 2016). Furthermore, short read RNAseq cannot provide information regarding concurrent alternative or rare splicing isoforms (Lee et al., 2021). There are six modes of alternative splicing (exon skipping, intron retention, constitutive splicing, mutually exclusive exons & alternative 3′ or 5’ splice sites) that are observed at varied frequencies in organisms, with intron retention most frequently detected in protozoa and lower metazoans, and exon skipping in higher metazoans (Wang et al., 2015; Yeoh et al., 2019). Long-read technologies such as Pacific Biosciences (PacBio) IsoSeq and Oxford Nanopore (ONT) direct RNA sequencing could be used to examine alternative splicing aiding in the verification and annotation of genes, and identification novel splice forms. In addition to limited information regarding splicing there are currently, no models for nucleotide modification in *Cryptosporidium* species and there are mixed reports on the evidence of methylation sites in the genome (Gissot et al., 2008; Aliaga et al., 2019). However, with the discovery of alternative methylation models in other apicomplexans (Baumgarten et al., 2019) it is important to reinvestigate and identify these models in *Cryptosporidium* species and ONT direct RNAseq will allow researchers to do this.

Long-RNA reads have already revealed several insights into the biology of related apicomplexan parasites, *Toxoplasma gondii* and *Plasmodium falciparum.* Transcripts from both *T. gondii* and *P. falciparum* retain large quantities of intronic sequences (Lee et al., 2021). Interestingly, in these parasites, intron retention rarely corresponds to expanded protein expression through alternative protein products, instead retention of introns primarily renders transcripts untranslatable and marked for nonsense mediated decay (NMD) (Lee et al., 2021). A detailed understanding of the transcriptional landscape in any *Cryptosporidium* species is outstanding. Putative alternatively spliced genes have been identified in *C. parvum* and alternative splicing regulators identified in *Cryptosporidium muris*, yet these splice forms and mechanisms are yet to be experimentally validated (Yeoh et al., 2019; Baptista et al., 2022).

## Discussion

The recent advances in sequencing technologies have expanded the capability of genomics and transcriptomics, and this can only be expected to increase as long-read sequencing is used on a population scale. Long read technologies not only increase resolution for haplotype collapsed reference genomes they can provide individual variant validation (Logsdon et al., 2020) and increased use of long read technologies on both these fronts will significantly aid *Cryptosporidium* research. Generating high quality reference genomes of more *Cryptosporidium* species will facilitate the identification of robust marker genes that can be used for MLST of isolates during general surveillance and clinical diagnosis (Morris et al., 2019). Individual variant resolution using long-read sequencing will help us characterise isolates, identifying genes and structural variants that drive pathogenicity. One example of where this will be a particularly useful tool is in the investigations of *C. tyzzeri.* The study by Sateriale et al. isolated a wild strain of the parasite that causes disease in laboratory mice that recapitulates human pathology (Sateriale et al., 2019). In contrast, Russler-Germain et al. identified a strain of *C. tyzzeri* that occurs commensally in laboratory mice (Russler-Germain et al., 2021). Genomic analysis of these variants (and others) may provide a genetic explanation for the observed differences in pathology.

In addition to facilitating better identification and characterisation of acute infections that can inform and improve patient care, sequencing can inform epidemiological policy more broadly through better parasite surveillance. Generating tools and methods for deconvoluting inter- and intra- specific *Cryptosporidium* population structure is challenging due to its compact genome, sexual recombination, CNV, and horizontal gene transfer. However, it is imperative that we continue to explore these questions in order to identify the mechanisms behind observations like increased transmissibility and virulence, reinfection, and seasonality, learning from long read sequencing studies in *Plasmodium* (Lin et al., 2018; Runtuwene et al., 2018; Yang et al., 2021). Studies in other Apicomplexa species should inform our increased use of long-read DNA sequencing in *Cryptosporidium* and subsequent data sets will facilitate the development of enhanced diagnostics arrays and accurate genotyping.

Long-read RNAseq technologies have and will continue to reveal important insights into the transcriptional landscape of the genome and improve our annotation and validation of genes (Lee et al., 2021; Baptista et al., 2022). Studying complete transcripts can help increase our understanding of the contents of the UTRs; for example, helping us identify promoter sequences that can be used for genetic studies, and may form potential therapeutic targets. Long-read transcriptomics can also shed light on the abundance of splice isoforms across the life cycle, thereby increasing our understanding of what defines each stage and the transitions between them. For example, NMD splicing was found to be prevalent in *T. gondii* and *P. falciparum* (Lee et al., 2021), and while NMD is typically thought to be stochastic (Saudemont et al., 2017), it can be regulatory, and in *Plasmodium* and *Toxoplasma* alternative splicing modulates transitions between life cycle stages (Yeoh et al., 2019; Lee et al., 2021). Long-read transcriptomics across the different stages of the *Cryptosporidium* life cycle will likely help us understand the mechanisms that drive sexual commitment, possibly unlocking methods to continuously propagate the parasite, *in vitro*.

*Cryptosporidium* research has been hindered in the past by the inconsistent availability and quality of genomic and transcriptomic data. The continuing advances in sequencing technologies, particularly with regards to long-read DNA and RNA sequencing, are perfectly positioned to drive forward *Cryptosporidium* research. The wider use of such technologies will increase our understanding of the parasite biology and facilitating translational advances in surveillance, diagnostics, and therapeutics.

## Author Contributions

TM and AS wrote this manuscript together. All authors contributed to the article and approved the submitted version.

## Funding

This work was supported by funding from The Francis Crick Institute (https://www.crick.ac.uk/), which receives its core funding from Cancer Research UK, the UK Medical Research Council and the Wellcome Trust.

## Conflict of Interest

The authors declare that the research was conducted in the absence of any commercial or financial relationships that could be construed as a potential conflict of interest.

## Publisher’s Note

All claims expressed in this article are solely those of the authors and do not necessarily represent those of their affiliated organizations, or those of the publisher, the editors and the reviewers. Any product that may be evaluated in this article, or claim that may be made by its manufacturer, is not guaranteed or endorsed by the publisher.
